# Experimental and Numerical Study on Mechanical Behavior of Steel/GFRP/CFRP Hybrid Structure under Bending Loading with Adhesive Bond Strength Assessment

**DOI:** 10.3390/ma16145069

**Published:** 2023-07-18

**Authors:** Jerzy Marszałek, Jacek Stadnicki

**Affiliations:** Department of Mechanical Engineering Fundamentals, Faculty of Mechanical Engineering and Computer Science, University of Bielsko-Biala, Willowa 2, 43-309 Bielsko-Biala, Poland; jstadnicki@ath.bielsko.pl

**Keywords:** hybrid structure, advanced high-strength steel, carbon- and glass-fiber-reinforced polymer composites, debonding, mechanical testing, finite element analysis

## Abstract

Adhesive bonding between steel and carbon-fiber-reinforced polymer (CFRP) composite leads to hybrid structures that combine the high strength and ductility of steel with the excellent specific strength and stiffness of CFRP composite. There is, however, a concern regarding possible galvanic corrosion when steel and carbon fibers are bonded together. One way to overcome this problem is placing glass fiber-reinforced polymer (GFRP) composite between the steel and CFRP composite, creating a more complex steel/GFRP/CFRP hybrid structure. Therefore, experimental and numerical studies on the mechanical behavior of the adhesive bonds between the steel sheet and the GFRP/CFRP hybrid composite were carried out. Among the different failure patterns, mode II was chosen for analysis because metal–polymer composite structures are usually subjected to bending, and debonding may occur due to in-plane shear stress. The tested steel/GFRP/CFRP hybrid structure was made of a hot-formed 22MnB5 boron steel sheet, intermediate single-ply bidirectional GFRP composite, and three-ply unidirectional CFRP composite. Additional mechanical tests were also carried out to determine various engineering constants of the components to simulate the debonding process. A finite element model of the steel/GFRP/CFRP hybrid structure with a typical cohesive interface was established and verified against the experimental data. The results showed that due to the use of various materials, the dominant failure modes in the hybrid structure under bending loading were a brittle fracture of the CFRP composite and debonding between the steel and the GFRP composite. However, the load-bearing capacity of the hybrid structure was five times greater than that of a non-reinforced steel sheet. In addition, its mass was only 28% greater than the non-reinforced steel sheet. The obtained results provided valuable conclusions and useful data to continue further research on the mechanical behavior of steel/GFRP/CFRP hybrid structures.

## 1. Introduction

Carbon-fiber-reinforced polymer (CFRP) composites have many attractive properties, such as high static and fatigue strength, high stiffness, low density and thermal expansion, and good corrosion resistance under various environmental conditions [1,2]. CFRP composites are particularly used in mechanical engineering due to their high strength-to-density ratio and are considered the standard construction material for new aerospace vehicles. These materials are also increasingly used in the automotive sector, and this use is expected to increase rapidly in the next two decades [3]. The main reason for the growing interest in CFRP composites in the automotive sector is their ability to reduce vehicles’ weight and thus improve fuel efficiency and reduce carbon dioxide emissions, while maintaining or even improving safety and mechanical performance [4,5]. The parts of passenger cars made from CFRP composites can be divided into two categories: parts improving the aesthetics of vehicles and load-bearing parts. The first group includes interior parts and finishes, front dashboard trims, interior and external trim cover panels, door handles, and side mirror covers. Such parts have no significant effect on the weight of the car and do not carry the load. The major reason for using CFRP composites in these locations is the attractive appearance resulting from the weave pattern of the carbon rovings in fabrics. The use of CFRP composites for load-bearing parts is aimed at reducing the weight of the vehicle while maintaining the required strength and stiffness. Therefore, these materials are used for larger parts of the vehicle structure, such as the monocoque chassis, side frames, and internal doorsill stiffening parts [6,7], while other parts are currently in the testing phase—for example, B-pillars [8]. It should be noted that in most cars, these parts are made of hot-formed (press hardening process) or cold-formed steels that ensure adequate stiffness and strength [9]. To obtain a similar mechanical performance for parts made from CFRP composites, carbon fibers with a high mechanical performance must be used, which significantly increase the cost of these parts. There are also some difficulties associated with the application of the composite (particularly to items with large dimensions and irregular shapes), such as intricate design procedures, high cost, and complex manufacturing processes [7]. All these reasons limit the use of CFRP composites in the construction of mass-produced passenger vehicles. The current use of parts made entirely of CFRP composites is restricted to niche segments of automobiles, such as luxury, premium, and sport cars [10]. The design of automotive lightweight structures can also be achieved using steel/CFRP hybrid structures. The high weight-reduction potential of steel/CFRP hybrid structures is obtained by an effective thickness reduction in the steel part with the simultaneous use of high mechanical performance CFRP composites [11]. Therefore, the application of steel/CFRP hybrid structures in the design and production of vehicle structures aims to increase the mass share of the lightweight and durable composite at the expense of the durable, but heavier, steel. Hybrid structures can also be used in another way, where the steel component of a vehicle body is locally reinforced with CFRP composite to improve its strength and stiffness with only a slight increase in weight.

The connection between the steel and composite is generally realized by adhesive bonding and mechanical fastening [12]. Adhesive bonding is inherently preferable to mechanical fastening because of the continuous connection formed. Composite materials exhibit a major decrease in their mechanical properties due to holes for joining using rivets or bolts because of the high notch sensitivity of these materials [13]. In addition, elements of mechanical fastenings, such as bolts or rivets, lead to a weight increase. Therefore, steel/CFRP hybrid structures, particularly when joined by an adhesive, have many advantages, and their use in automotive applications has great potential.

Despite their many advantages, steel/CFRP hybrid structures are currently only used to a limited extent in mass-produced passenger vehicles. According to the literature, there have not been many attempts to use steel/CFRP hybrid structures in the design of automotive parts, particularly those with large sizes and complicated shapes. An example is the research concerned with vehicle structural components, such as the center-pillar [14,15,16,17]. The limited use is due to the longer manufacturing lead-time compared with manufacturing steel components and difficulties in the measurement of the mechanical properties of these materials. The mechanical behavior of steel/CFRP hybrid structures depends not only on the properties of their components but also on the properties of the adhesive joint. As is well known, the mechanical behavior of fiber-reinforced polymers also depends on the manufacturing conditions, such as the manufacturing technique and curing cycle. For this reason, investigations on steel/CFRP hybrid structures are still at the basic experimental stage, and focus on their mechanical properties, adhesive joint strength, and formability [17].

An additional difficulty in using steel/CFRP hybrid structures in engineering applications is the possibility of galvanic corrosion, which can occur due to steel and CFRP composites having very different electrical potentials [18]. Extensive galvanic corrosion of steel in contact with carbon fibers particularly occurs in aggressive environments, such as aqueous solutions containing salts, acids, or fuel combustion products [19]. Generally, corrosion propagation can lead to failure of the adhesively bonded joint in steel/CFRP hybrid structures. A basic method for suppressing galvanic corrosion is to have no contact between the carbon fibers and steel, so the adhesive layer alone can be effective in suppressing the potential galvanic corrosion of the steel/CFRP hybrid structure, and embedding glass fiber ply in the adhesive can additionally increase this effect [18]. The application of additional glass fiber for increasing the protection against corrosion leads to a three-phase hybrid structure, which includes steel sheet, CFRP composite, and glass-fiber-reinforced polymer (GFRP) composite. This type of hybrid structure can be abbreviated as steel/GFRP(m)/CFRP(n), where the parameters m and n indicate the number of plies in the GFRP and CFRP composites, respectively.

To apply any material in engineering practice, it is necessary to study its key mechanical properties, such as strength and stiffness, under various load conditions. It is well known that steel and many types of polymer composites have sufficient tensile strength for most engineering applications. A particularly important mechanical property of steel/polymer composite hybrid structures intended for the automotive industry is the shear adhesive bond strength, because automotive components are generally thin-walled, and the dominant loading condition is bending. When a steel/polymer composite hybrid structure is subjected to bending, sliding shear and in-plane shear stresses are observed between the steel sheet and polymer composite, giving a risk of debonding formation and propagation, which may result in the complete destruction of the adhesive bond between the steel sheet and polymer composite.

Despite the considerable number of studies in the literature about the debonding failure of the steel/CFRP(n) adhesive joint [20,21,22,23,24,25,26,27], few systematic studies on the bond behavior between steel sheets and GFRP(m) polymer composites have been performed, and a very limited number of papers have been published that focus on this topic [28,29,30,31]. To the best knowledge of the authors, no research has been published on the mechanical behavior of the adhesive bonds between steel sheets and GFRP(m)/CFRP(n) hybrid composites under three-point bending. Therefore, the primary objective of this research was experimental examination of the bending response of steel/GFRP(m)/CFRP(n) hybrid structure specimens with a particular focus on the possibility of debonding between the steel sheet and the GFRP(m)/CFRP(n) hybrid composite. The purpose of this study was also to develop a numerical model of the steel/GFRP(m)/CFRP(n) hybrid structure. The simulation results were a valuable supplement to the experimental research. Experimental and numerical analysis of the mechanical properties of steel/GFRP(m)/CFRP(n) hybrid structures requires data on the mechanical properties of their individual components. Therefore, in the current study, additional tests and numerical simulations were carried out to determine the material properties of the individual components.

The remainder of this paper is organized as follows. In Section 2, all the materials used in this study are characterized. Section 3 presents the experimental procedures and details of the finite element (FE) models employed, while the experimental and numerical results are discussed in Section 4. Finally, in Section 5, conclusions are drawn, and a summary is presented.

## 2. Materials

**Figure 1:**
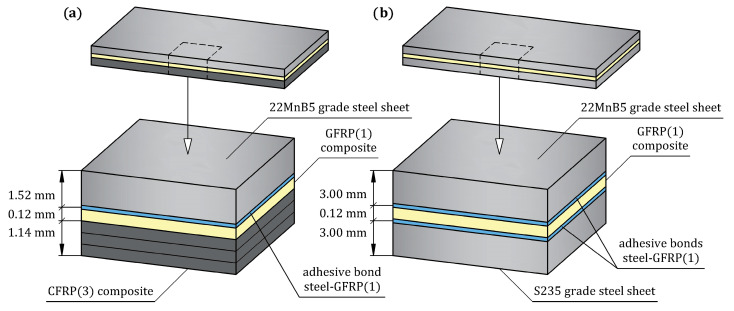
Schematic diagrams of hybrid structures investigated in this study: (**a**) steel/GFRP(1)/CFRP(3) hybrid structure; (**b**) steel/GFRP(1)/steel hybrid structure.

**Figure 2:**
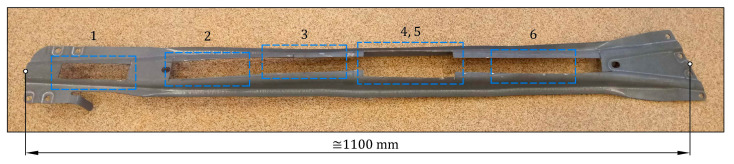
A side door beam of a passenger car and locations of cutting specimens for testing.

The experimental steel/GFRP(m)/CFRP(n) hybrid structure used in this research contained hot-formed 22MnB5 (1.5528) grade automotive steel, single-ply bidirectional GFRP composite (m = 1), and three-ply unidirectional CFRP composite (n = 3). The sequence of the individual materials of the steel/GFRP(1)/CFRP(3) hybrid structure with their average thickness are shown in Figure 1a. Preliminary experimental studies showed that the application of a CFRP(n) composite with a greater number of plies (n > 3) just slightly increased the bending bearing capacity of the hybrid structure. This result is related to the greater thickness of the CFRP composite and the faster achievement of the breaking stress in the layers subjected to tension during the bending test. As mentioned, the basic adherents were a 22MnB5 grade steel sheet and CFRP(3) composite. In turn, the GFRP(1) composite was used as intermediate material between the steel sheet and CFRP(3) composite to increase protection against galvanic corrosion. As shown in Figure 2, 22MnB5 grade steel sheets used to prepare steel/GFRP(1)/CFRP(3) hybrid structure specimens were cut off by a laser from flat surfaces of the hot-formed side door beam of a passenger car to take account of actual production conditions. The chemical composition of 22MnB5 grade steel in accordance with the DIN EN 10083-3 standard [32] is given in Table 1.

**Figure 3:**
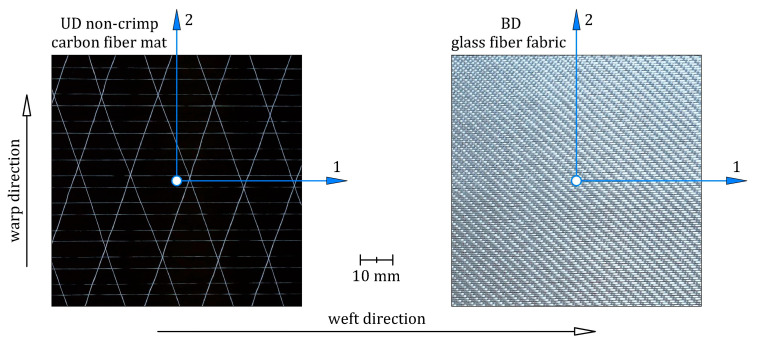
Photographs of the unidirectional non-crimp carbon fiber mat and bidirectional 2 × 2 twill glass fiber fabric.

The unidirectional CFRP(3) and bidirectional GFRP(1) composites were made from three plies of a unidirectional (UD) non-crimp carbon fiber mat and bidirectional (BD) glass fiber fabric, respectively (Figure 3). Details of the carbon fiber mat and glass fiber fabric selected for this study are listed in Table 2. The principal coordinate system of the reinforcements was a coordinate system with the 1-axis aligned with the fibers in the UD carbon mat and aligned with the weft in the BD glass fabric and the 2-axis perpendicular to the fibers in the UD carbon mat and aligned with the warp in the BD glass fabric. The epoxy resin (Biresin^®^ CR122) and hardener (Biresin^®^ CH122-5), mixed at a weight ratio of 100:30, were used both as the matrix material in the CFRP(3) and GFRP(1) composites and as the adhesive bonding between the steel sheet and GFRP(1) composite. Selected properties of the epoxy matrix are given in Table 3 according to the manufacturer’s datasheet. In this study, additional specimens of the hybrid structure, abbreviated as steel/GFRP(1)/steel, were used to investigate the mode II fracture toughness of the adhesive bond between the steel adherent and GFRP(1) composite. The adherents were made of S235 grade steel, the chemical composition of which is given in Table 4. The sequence of the individual materials of the steel/GFRP(1)/steel hybrid structure with their average thickness are shown in Figure 1b. All materials used in this study were supplied by local suppliers.

## 3. Experimental and Numerical Procedures

### 3.1. Experimental Tests

A comprehensive experimental program was carried out in this study consisting of three-point bending tests on steel/GFRP(1)/CFRP(3) hybrid structure specimens and additional mechanical tests, including tensile and bending tests on 22MnB5 grade steel specimens, tensile tests on CFRP and GFRP composite specimens, and debonding of the adhesive joint between GFRP and steel in steel/GFRP(1)/steel hybrid structure specimens. The experiments were performed using Inspekt Table Blue 5 (Hegewald & Peschke, Nossen, Germany) and Instron 68FM-300 (Instron, Norwood, MA, USA) universal testing machines under standard laboratory conditions with a temperature of 23 ± 2 °C and relative humidity of 50 ± 10%.

#### 3.1.1. Tensile Test on 22MnB5 Grade Steel Specimens

The tensile properties of 22MnB5 grade steel had been determined as a part of previous investigations and had been presented in Reference [34]. The results of these investigations were used in the current study to define a non-linear material model of 22MnB5 grade steel for FE simulations. Elastic-plastic properties of 22MnB5 grade steel were measured by a uniaxial tensile test according to the ISO 6892-1:2009 A224 standard [35]. An extensometer of 80 mm gauge length was attached to the center of the specimen to measure the longitudinal strain. The tensile velocity was set to 1 mm/min. To ensure repeatability, four specimens were tested. The specimens were cut from the flat surfaces of a final automotive component made by the hot-forming process.

**Figure 4:**
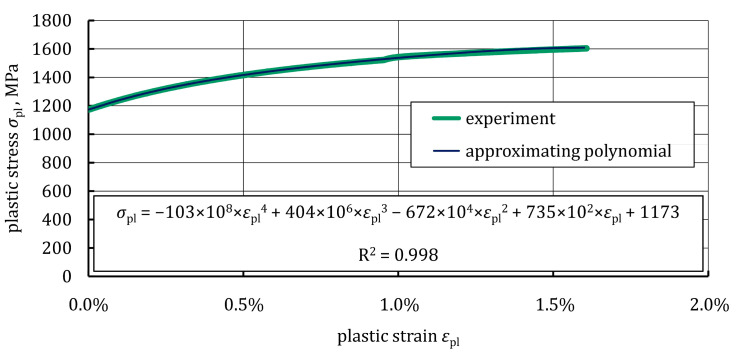
Average plastic deformation curve with approximating polynomial.

Based on the average stress-strain curve, the conventional yield strength and the ultimate tensile strength of the 22MnB5 grade steel were equal to 1173 MPa and 1615 MPa, respectively. Very similar tensile properties of hot-formed 22MnB5 grade steel, after its complete austenitization, are presented in References [36,37,38]. To accurately describe the plastic behavior of the 22MnB5 grade steel, the experimental plastic curve, *σ*_pl_(*ε*_pl_), was approximated by a four-degree polynomial, as shown in Figure 4.

#### 3.1.2. Three-Point Bending Test on 22MnB5 Grade Steel Specimens

**Figure 5:**
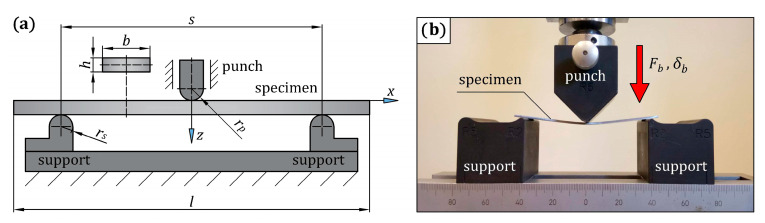
(**a**) Three-point bending test setup on a 22MnB5 grade steel specimen with its geometry and dimensions; (**b**) the specimen placed on the three-point bending fixture.

Experimental bending tests on the 22MnB5 grade steel specimens were carried out for two reasons: the results were used to compare the bending response of non-reinforced steel with the bending response of steel/GFRP(1)/CFRP(3) hybrid structure specimens, while the simulation of the bending test on non-reinforced steel allowed the correctness of the elastic-plastic material model of steel determined in the tensile test to be checked. The scheme of the bending stand and 22MnB5 grade steel specimen are shown in Figure 5a, and their dimensions are listed in Table 5. The quasi-static three-point bending test was performed using the bending fixture in the Inspekt Table Blue testing machine. A total of three specimens were tested to ensure repeatability. As mentioned in Section 2, the specimens were cut from an automotive side door beam. The specimens were placed on the bending fixture with two semicylindrical steel supports and bent by the middle semicylindrical steel punch, as shown in Figure 5b. During the test, a deflection, *δ_b_*, equivalent to the vertical displacement of the punch, was applied at the midspan with a rate of 1 mm/min, and the reaction force, *F_b_*, experienced by the specimen was measured. The values of *δ_b_* and *F_b_* were recorded during the loading process for the evaluation of the *F_b_*(*δ_b_*) curve for each specimen.

#### 3.1.3. Tensile Test on Single-Ply CFRP and GFRP Composite Specimens

**Figure 6:**
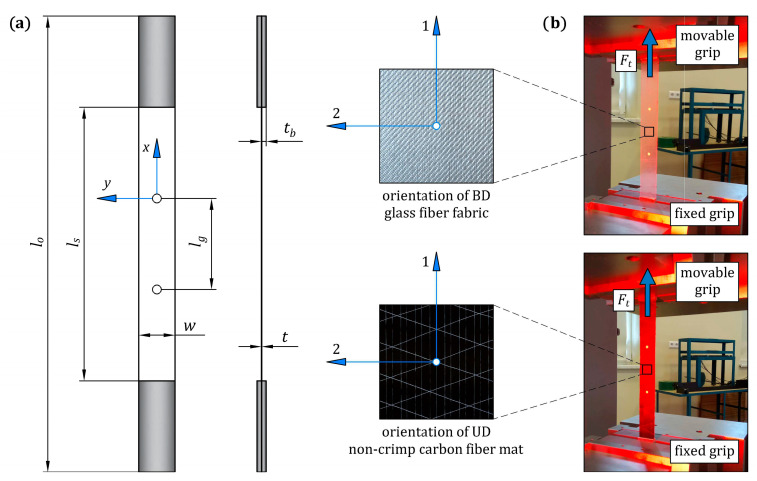
Uniaxial tensile test of the single-ply CFRP and GFRP composite specimens: (**a**) dimensions of the specimens; (**b**) experimental setup for testing.

**Figure 7:**
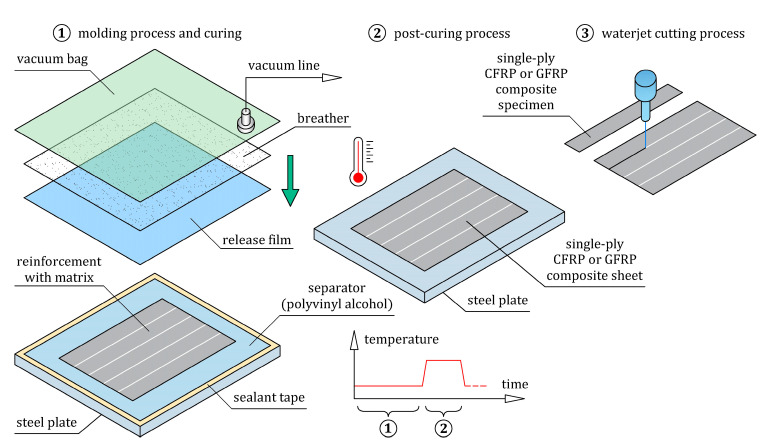
Schematic illustration of the preparation of single-ply CFRP and GFRP composite specimens.

Uniaxial tensile tests were conducted to determine the homogenized longitudinal tensile elastic modulus, $E_{11}^{T}$, and the ultimate tensile strain, $\varepsilon_{UTS}$, of the single-ply CFRP and GFRP composites. The tensile test was conducted based on the ASTM D3039 standard [39], which is dedicated to polymer matrix composite materials. The geometry and dimensions of the tensile test specimens are presented in Figure 6a, and their nominal dimensions are given in Table 6. The *x*-*y* coordinate system was used for the specimens’ coordinates, where the *x*-axis and *y*-axis indicated longitudinal and transverse directions, respectively. In both CFRP and GFRP composite specimens, the 1-axis of the reinforcement was parallel to the *x*-axis, which was consistent with the direction of applied tensile load. The specimens were cut from previously prepared composite sheets, which were fabricated using the vacuum bagging fabrication method under conditions including curing at room temperature for 24 h and post-curing cycle (1 °C/min ramp up to 80 °C, 5 h isothermal at 80 °C, 1 °C/min ramp down to room temperature), as shown in Figure 7. The composite sheets were cut by a waterjet to ensure the final dimensions of the specimens were correct. A total of three specimens of each composite type were prepared and tested. Aluminum end-tabs were glued to the ends of all specimens to minimize the stress concentration near the grips of the tensile testing machine. Two white marker points were painted on the surface of each specimen at a distance equal to the gauge length. A non-contact video extensometer to evaluate the strain along the gauge length was used in this experiment. The region where the strain was measured during the tensile test is visible in Figure 6b. The tensile tests were performed with the Instron 68FM-300 universal testing machine under displacement control with a crosshead speed of 1 mm/min. For each specimen, the longitudinal local strain, $\varepsilon_{11}^{T}$, was extracted from video extensometer measurements, and the longitudinal local tensile stress, $\sigma_{11}^{T}$, was determined by dividing the tensile force, *F_t_*, by the initial cross-section of the specimen. The homogenized longitudinal tensile elastic modulus, $E_{11}^{T}$, and the ultimate tensile strain, $\varepsilon_{UTS}$, for each tested specimen were determined from the experimental stress-strain curve, $\sigma_{11}^{T}\left(\varepsilon_{11}^{T}\right)$. The modulus, $E_{11}^{T}$, is defined as the slope of the stress-strain curve, $\sigma_{11}^{T}\left(\varepsilon_{11}^{T}\right)$, in the interval between the two strains $\varepsilon_{11(1)}^{T}=0.05\%$ and $\varepsilon_{11(2)}^{T}=0.25\%$.

#### 3.1.4. End-Notched Flexure Test on Steel/GFRP(1)/Steel Hybrid Structure Specimens

**Figure 8:**
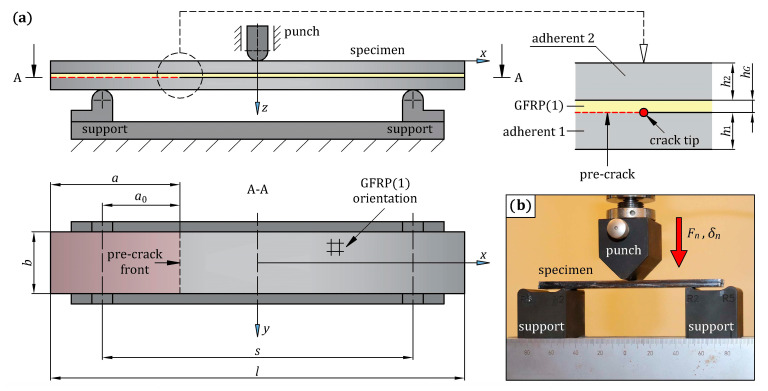
(**a**) Geometry and dimensions of the steel/GFRP(1)/steel hybrid structure specimen used for the ENF test; (**b**) the specimen in three-point bending fixture.

The three-point bend end-notched flexure (ENF) test, in combination with FE analysis, was used to examine the mode II fracture toughness of the adhesive bond between the steel adherents and the single-ply GFRP composite. The scheme of the stand and the specimen for the ENF test are shown in Figure 8a, and the dimensions of them are listed in Table 7. The specimen consisted of two S235 grade steel adherents with single-ply GFRP composite placed between them, as shown in Figure 1b. S235 grade steel was used to prepare the ENF specimen because it was easily available in sheets of any thickness, whereas hot-formed 22MnB5 grade steel sheets with high thickness were less accessible. This was important because the thickness of the ENF specimens had to be large enough to cause break initiation in the adhesive bond between the steel and GFRP(1) composite before the ultimate strain occurred in the steel during the test. It was necessary to ensure that the surface roughness of the S235 grade steel sheets was the same as the 22MnB5 grade steel specimens cut off the hot-formed side door beam. The required surface roughness (Ra = 1.25 μm) was obtained by sandblasting. Both the hand lay-up technique and compression molding were used to manufacture three specimens designated as steel/GFRP(1)/steel.

**Figure 9:**
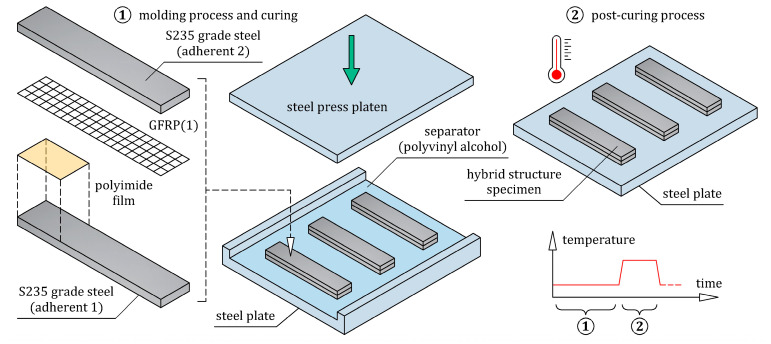
Fabrication process of steel/GFRP(1)/steel hybrid structure specimens.

Each specimen was prepared separately under the same manufacturing conditions (Figure 9). Firstly, the steel adherents were cleaned and degreased, after which one of them (adherent 1) was placed in the mold. On the upper surface of adherent 1, a thin matrix layer was evenly distributed. As mentioned in Section 2, the matrix was prepared as a mixture of epoxy resin and hardener in a weight ratio of 100:40. An initial pre-crack at one end of the specimen was made by inserting a 12.5 μm thick polyimide film on the adherent 1. One layer of bidirectional 2 × 2 twill weave glass fiber fabric impregnated with the matrix was then applied to the matrix-coated adherent 1. The fabric was placed on the adherent 1 in a 0/90 orientation, meaning that the 1–2 coordinate system of the fabric (Figure 3) coincided with the *x*-*y* coordinate system of the specimen (Figure 8a). Finally, the glass fiber fabric was covered with the one-sided matrix-coated adherent 2. After the hand lay-up, the steel/GFRP(1)/steel hybrid structure was pressed by the compression plate, which applied a pressure of 0.01 MPa. The curing process of the specimen was divided into two steps. Firstly, the specimen was cured at room temperature for 24 h under pressure, after which the specimen was taken out from the mold and post-cured according to the cycle: 1 °C/min ramp up to 80 °C, 5 h isothermal at 80 °C, 1 °C/min ramp down to room temperature. An ENF test of the three steel/GFRP(1)/steel hybrid structure specimens was carried out with the Inspekt Table Blue universal testing machine. Figure 8b shows the specimen in the test fixture. The specimens were loaded at a crosshead speed of 1 mm/min until the crack tip obviously passed the midspan. All tests were performed under deflection control, *δ_n_*, and the vertical reaction force, *F_n_*, experienced by the specimen was measured. The pre-crack length, *a*_0_, was defined as the distance from the support to the crack tip (Figure 8a).

#### 3.1.5. Three-Point Bending Test on Steel/GFRP(1)/CFRP(3) Hybrid Structure Specimens

**Figure 10:**
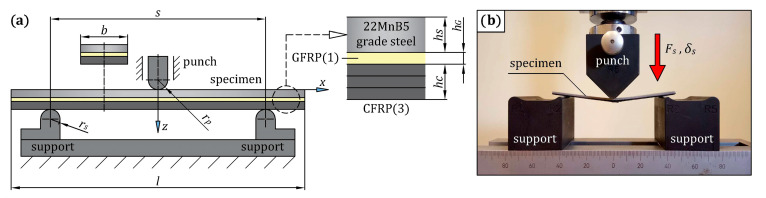
(**a**) Three-point bending test setup on steel/GFRP(1)/CFRP(3) hybrid structure with its geometry and dimensions; (**b**) specimen placed on the three-point bending fixture.

The main experiment in the current study was to investigate the stiffness and strength of the steel/GFRP(1)/CFRP(3) hybrid structure specimens under bending loading. The bending test was performed in a three-point configuration on rectangular specimens, and the failure mechanism was investigated by visual inspection. The scheme of the bending stand and the specimen are shown in Figure 10a, and their dimensions are listed in Table 8. To ensure the reliability of the test results, three specimens were selected for the test, and the average value was taken to be analyzed. Each specimen underwent the three-point bending test on the Inspekt Table Blue universal testing machine, as shown in Figure 10b. The loading rate was 1 mm/min, and the deflection, *δ_s_*, and force, *F_s_*, data were measured by sensors in the loading device of the test machine. During the test, the CFRP(3) composite was under tension, while the steel sheet was subjected to compression.

**Figure 11:**
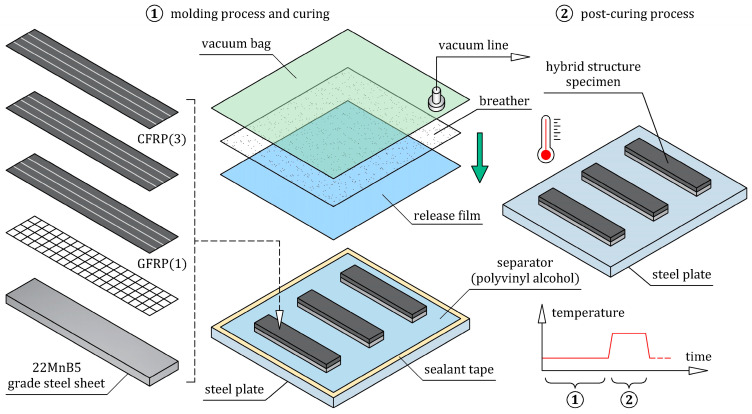
Fabrication process of steel/GFRP(1)/CFRP(3) hybrid structure specimens.

Steel/GFRP(1)/CFRP(3) hybrid structure specimens were fabricated at the same time by the wet lay-up/vacuum bag method without applying any extra pressure (the specimens were only compacted by the vacuum pressure). The typical stages of manufacturing are schematically presented in Figure 11. Firstly, the polished steel base was cleaned and covered by a layer of release film for easy removal of the manufactured specimens. Three steel sheets with dimensions of 100 mm × 20 mm, which were cut from the hot-formed side door beam of a passenger car (Figure 2), were cleaned and placed on the steel base. On the upper surface of each steel sheet, a thin matrix layer was evenly distributed. One layer of bidirectional 2 × 2 twill weave glass fiber fabric with dimensions of 105 mm × 25 mm was placed in a 0/90 orientation to each steel sheet. Finally, three layers of unidirectional (UD) non-crimp carbon fiber mat with dimensions of 105 mm × 25 mm were applied longitudinally to each steel/GFRP(1) stack. The entire assembly was covered in a vacuum bag. The curing process of the specimens was divided into two steps, as applied to the steel/GFRP(1)/steel hybrid structure. Firstly, the specimen was cured at room temperature for 24 h in a vacuum bag, after which the specimen was post-cured (cycle: 1 °C/min ramp up to 80 °C, 5 h isothermal at 80 °C, 1 °C/min ramp down to room temperature). The excess of the GFRP(1)/CFRP(3) hybrid composite projecting beyond the side edges of the steel sheet was removed by grinding.

### 3.2. FE Modeling

FE modeling was used to perform numerical simulations of the following experiments:

- three-point bending test on the 22MnB5 grade steel specimen. The results of this simulation were compared with the experimental results to verify the accuracy of the non-linear material model of 22MnB5 grade steel determined experimentally in the tensile test.
- ENF test on the steel/GFRP(1)/steel hybrid structure specimen. This simulation was used to determine the parameters of the cohesive zone model (CZM) in the bilinear traction-separation law for the correct modeling of the adhesive bond between the steel and GFRP(1) composite.
- three-point bending test on steel/GFRP(1)/CFRP(3) hybrid structure specimen. The results of this simulation supplemented the experimental studies in which the stiffness and strength of the steel/GFRP(1)/CFRP(3) hybrid structure were tested under three-point bending loading conditions.

**Figure 12:**
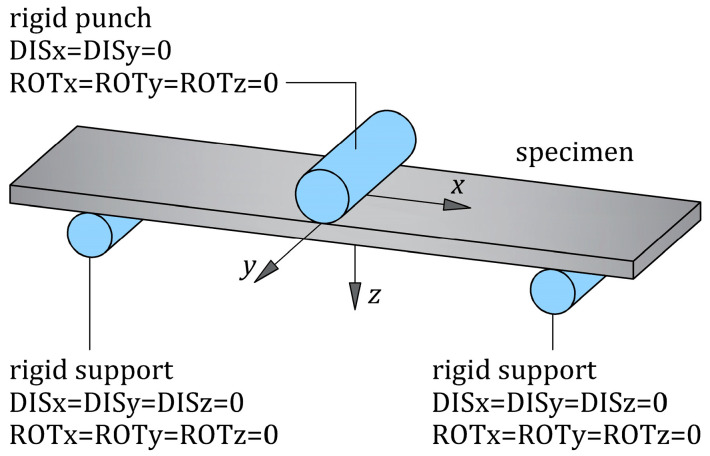
Boundary conditions for the specimen during three-point bending loading, where DISx and ROTx mean displacement along the *x*-axis and rotation around the *x*-axis, respectively (similarly for the other axes).

Numerical simulations of the tests were performed with Siemens Simcenter 3D software (version 1996) using the advanced non-linear solver NX Nastran (SOL 601). To accurately simulate the behavior of the specimens in FE analysis, the dimensions of the specimens, the orientation of the composite plies, the radii of the punch and supports, and the span length were the same as those in the experiments. The punch and supports were modeled as rigid bodies. Boundary conditions are presented in Figure 12. The deflection was realized by a movable punch at the mid-span of the specimens, and the reaction force of the specimens was computed. The model of each specimen is described in detail below.

#### 3.2.1. Simulation of Three-Point Bending Test on 22MnB5 Grade Steel Specimen

**Figure 13:**
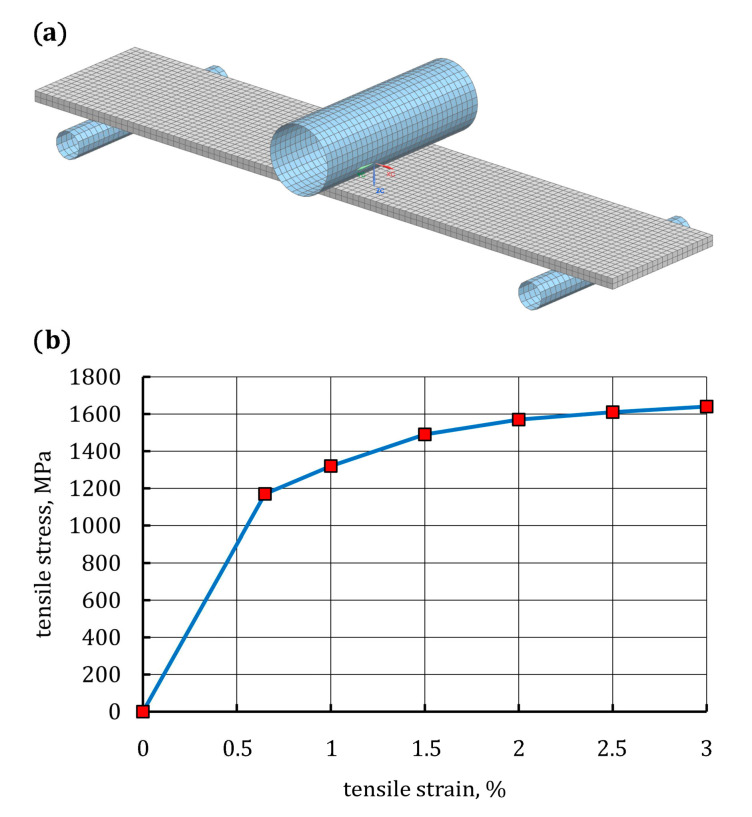
(**a**) FE model of the 22MnB5 grade steel specimen for the three-point bending test; (**b**) multilinear isotropic hardening model of 22MnB5 grade steel used in FE analysis.

As mentioned above, an FE model was used to simulate the bending response of the 22MnB5 grade steel specimen considering the non-linear stress-strain relationship of the steel determined in the tensile test. A three-dimensional FE model for the three-point bending test of 22MnB5 grade steel specimen is shown in Figure 13a. The elastic-plastic specimen was modeled with continuum eight-node hexagonal solid elements (T8). The non-linear stress-strain relationship of the steel derived from the tensile test was programmed as a multilinear isotropic hardening model, as shown in Figure 13b. The stress-strain relationship was limited to 3% because it was sufficient to simulate the deflection of the specimen during the bending test. As can be seen in Figure 13a, the specimen geometry and loading condition in the FE simulations were the same as those applied in the bending test. In the contact between the punch, the supports, and the surfaces of the specimen, friction was considered, and the friction coefficient was 0.5 [40]. The deflection was constrained at the nodes of the punch, and a maximum deflection of 12 mm was applied on the midspan.

#### 3.2.2. Micro-Mechanics Modeling

**Figure 14:**
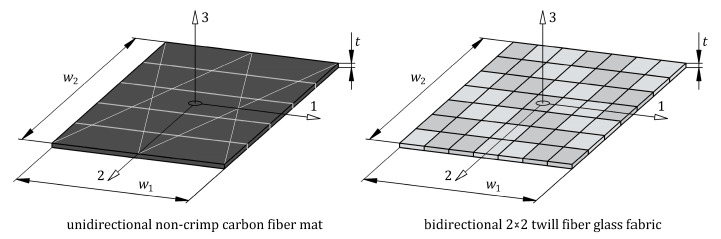
Schematic illustration of single-ply CFRP and GFRP composites with orthotropic axes.

The CFRP and GFRP composites included in the steel/GFRP(1)/CFRP(3) hybrid structure exhibit orthotropic behavior. The constitutive law for a tridimensional orthotropic material consists of nine independent material constants: *E*_11_, *E*_22_, *E*_33_, *G*_12_, *G*_13_, *G*_23_, *v*_12_, *v*_13_, and *v*_23_. The elastic moduli, *E*_11_, *E*_22_, and *E*_33_, are commonly obtained from appropriate tensile tests performed along the main axes of orthotropy (in directions 1, 2, and 3, respectively) as shown in Figure 14. Determination of the in-plane shear modulus, *G*_12_, the out of plane shear moduli, *G*_13_ and *G*_23_, the in-plane Poisson’s ratio, *v*_12_, and thickness Poisson’s ratios, *v*_13_ and *v*_23_, requires a combination of many experimental tests. In this present study, all these material constants of CFRP and GFRP composites were determined with the use of a modular NX simulation toolset for laminate composite structures. Micromechanical models were implemented in the program, which were used to generate composite material constants based on the following inputs: elastic modulus of fibers (*E_f_*) and matrix (*E_m_*), Poisson’s ratio of fibers (*v_f_*) and matrix (*v_m_*), and volume fraction of fibers (*V_f_*) and matrix (*V_m_*). Based on the data available from the literature, the Poisson’s ratio of carbon and glass fibers is equal to 0.2 and 0.17, respectively [41,42]. In this present study, the matrix volume fraction, *V_m_* (percentage by volume), of single-ply CFRP and GFRP polymer composites was calculated according to the following equation

(1) $$V_{m}=100\times \left(\frac{m_{cs}-m_{rf}}{\rho_{m}}\right)\times \frac{1}{V_{cs}}$$

where *m_cs_* is the mass of single-ply polymer composite with dimensions of *w*_1_ × *w*_2_ × *t*, *m_rf_* is the mass of dry reinforcement fabric with dimensions of *w*_1_ × *w*_2_, *ρ_m_* is the density of the matrix, and *V_cs_* is the volume of the single-ply polymer composite with dimensions of *w*_1_ × *w*_2_ × *t* (Figure 14). The density of the matrix, *ρ_m_*, and the mass of dry reinforcement fabric, *m_rf_*, are usually available from the manufacturer’s datasheets. The fiber volume fraction, *V_f_* (percentage by volume), was determined using the following equation

(2) $$V_{f}=1-V_{m}$$

The unknown elastic modulus of the fibers, *E_f_*, was estimated by combining a theoretical and experimental approach. The method is based on the generalized rule of mixture and the results of the axial tensile test on a single-ply composite specimen. The generalized rule of mixture is expressed as

(3) $$E_{11}^{T}=\eta_{L}\eta_{o}E_{f}V_{f}+E_{m}\left(1-V_{f}\right)$$

where *η_L_* is the length correction factor (*η_L_* ≈ 1 for fibers longer than 10 mm), while *η_o_* is the correction factor for non-unidirectional reinforcement (*η_o_* ≈ 1 for unidirectional reinforcement, *η_o_* ≈ 0.5 for balanced bidirectional reinforcement). To evaluate the elastic modulus of fibers, *E_f_*, the following equation can be used:

(4) $$E_{f}=\frac{E_{11}^{T}-E_{m}\left(1-V_{f}\right)}{\eta_{L}\eta_{o}V_{f}}$$

The tensile elastic modulus, $E_{11}^{T}$, of a polymer composite is commonly determined experimentally from the slope of a stress-strain curve created during uniaxial tensile testing. The elastic modulus of the matrix, *E_m_*, is usually available from the manufacturer’s datasheet.

#### 3.2.3. Simulation of ENF Test on Steel/GFRP(1)/Steel Hybrid Structure Specimen

**Figure 15:**
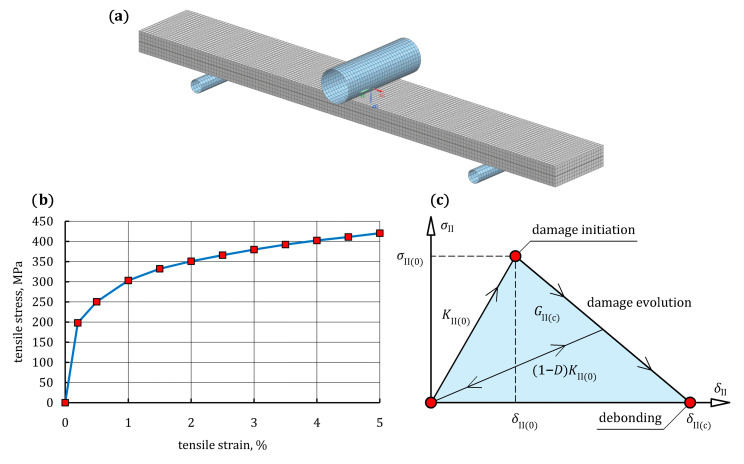
(**a**) FE model of the steel/GFRP(1)/steel hybrid structure specimen in the ENF test fixture; (**b**) multilinear isotropic hardening model of the S235 grade steel used in the FE analysis; (**c**) damage evolution curve for the bilinear cohesive element.

The geometry of the FE model for the steel/GFRP(1)/steel hybrid structure specimen with boundary conditions of the ENF test is shown in Figure 15a. In the FE model, the specimen consisted of five components, the lower and upper steel adherents (adherents 1 and 2, respectively), the intermediate single-ply GFRP composite, and two layers of cohesive elements. Both the steel adherents and the single-ply GFRP composite were modeled with continuum eight-node hexagonal solid elements (T8). Based on the data available from the literature [43], the stress-strain relationship of the S235 grade steel adherents was programmed as a multilinear isotropic hardening model (Figure 15b). The single-ply GFRP composite was considered as an orthotropic material. For the adhesive bond joint, eight-node cohesive elements (T147) with a bilinear traction-separation law were adopted to simulate debonding between the steel adherents and the single-ply GFRP composite. Cohesive elements were created using automatic meshing tools available in the NX Siemens software. This automatic meshing generated cohesive elements between the source (adherents) and the target (single-ply GFRP composite) faces. Due to a 50 mm long pre-crack, the first layer of cohesive elements was inserted between adherent 1 and the single-ply GFRP composite on the 100 mm length. The second layer of cohesive elements was inserted between adherent 2 and the single-ply GFRP composite along the entire length of the specimen (on the 150 mm length). The contact elements were inserted along the pre-crack. These contact elements were only able to transmit normal compressive stress, and friction effects were neglected.

A bilinear CZM was employed to simulate the debonding process. According to Figure 15c, the inputs for the bilinear traction-separation law, *σ*_II_(*δ*_II_), for mode II (sliding) are initial stiffness, *K*_II(0)_, interface shear strength, *σ*_II(0)_, and critical strain energy release rate, *G*_II(c)_ (damage initiation separation, *δ*_II(0)_, and final failure separation, *δ*_II(c)_, are resultant variables). The bilinear traction-separation law consists of an initial linear elastic stage until a damage initiation criterion is satisfied. The initial stiffness, *K*_II(0)_, that relates traction to the separation of a cohesive element before damage initiation is defined as below

(5) $$K_{II(0)}=\frac{\sigma_{II(0)}}{\delta_{II(0)}}$$

When traction (interface shear) at the crack tip reaches its maximum value *σ*_II(0)_, a linear softening phase occurs and the progressive decohesion of the interface with increasing damage is simulated. Finally, the traction declines to zero at *δ*_II(c)_ and new cracks are generated. The softening relation of cohesive elements that are governed by bilinear constitutive law can be expressed as

(6) $$\sigma_{II}=\left(1-D\right)K_{II\left(0\right)}\delta_{II}$$

where *D* is a variable that relates to the damage condition, which has a magnitude *D* = 0 when the interface is undamaged, and a magnitude *D* = 1 when the interface is completely fractured. Interpretation of the two adjacent layers after complete debonding is prevented by the modeling of the contact properties. The critical strain energy release rate, *G*_II(c)_, is defined as the area under the bilinear traction-separation characteristic. A more detailed description of the cohesive zone FE technique is presented, among others, in References [44,45,46].

The necessary parameters of the bilinear traction-separation law were determined in the calibration of the FE model. Firstly, the initial value of *K*_II(0)_ = 4400 N/mm^3^ was adopted because the linear region of the load-deflection curve of the model showed the best fitting to the linear region of the load-deflection curve from the experiment. To determine values of *σ*_II(0)_ and *G*_II(c)_, a special script was developed, which automatically ran successive simulations of the ENF test. Each time, the script changed the values of *σ*_II(0)_ and *G*_II(c)_ slightly and examined the response of the model. For each set of values of the *σ*_II(0)_ and *G*_II(c)_, the script compared the computational and experimental load-deflection curves of the specimen. Values of *σ*_II(0)_ and *G*_II(c)_ were searched until the non-linear region of the computational load-deflection curve was satisfactorily fitted to the experimental curve. Between the contact of the loading nose, the supports, and the surface of the specimen, friction was considered, the friction coefficient being 0.5 [40]. The vertical displacement was constrained at the nodes of the punch, and a maximum deflection of 1 mm was imposed on the midspan.

#### 3.2.4. Simulation of the Three-Point Bending Test on the Steel/GFRP(1)/CFRP(3) Hybrid Structure Specimen

**Figure 16:**
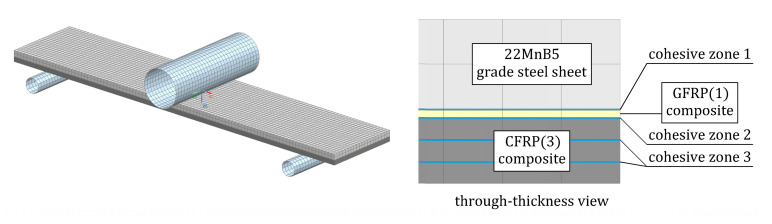
FE model of the steel/GFRP(1)/CFRP(3) hybrid structure specimen in the three-point bending test fixture.

The FE model used to simulate the bending response of the steel/GFRP(1)/CFRP(3) hybrid structure specimen tested in this study is shown in Figure 16. Each layer of the hybrid structure was considered as a homogeneous component and was connected by cohesive elements to the adjacent layers to simulate possible debonding and delamination. Eight-node hexagonal solid elements (T8) were used to model the steel sheet and individual plies of the GFRP(1)/CFRP(3) hybrid composite. Steel sheet behavior was computed using a multilinear isotropic hardening material model with a tensile yield limit, which was determined experimentally (Figure 4). The mechanical properties of GFRP(1) and CFRP(3) composites plies were obtained using the procedure described in Section 3.2.2 and implemented into the model as orthotropic linear elastic materials. Eight-node cohesive elements were spread out between the individual components at the bonded surfaces (on the 100 mm length). Cohesive elements (T147) governed by the bilinear traction-separation law were adopted to create three types of cohesive zones, i.e., cohesive zone 1 between the steel sheet and the GFRP(1) composite, cohesive zone 2 between the GFRP(1) and CFRP(3) composites, and cohesive zone 3 between the individual plies of the CFRP(3) composite, as shown in Figure 16. The parameters of the traction-separation law for cohesive zone 1 were determined based on the ENF test simulation and calibration of the FE model (Figure 15). To describe the cohesive behavior of the interface in zones 2 and 3, literature data were used [47,48]. In the contact between the supports and the CFRP composite, friction was considered, and the friction coefficient was 0.1 [49].

In accordance with well-known rules, during bending, the bottom surface of a beam is under tension, while the top surface is under compression, so during the three-point bending test of steel/GFRP(1)/CFRP(3) hybrid structure specimens, the GFRP(1)/CFRP(3) hybrid composite is particularly subject to tension. Thus, the ultimate tensile strain criterion was used in the simulation to predict the tensile failure of the GFRP(1) and CFRP(3) composites. According to ultimate tensile strain theory, the fracture of the single-ply composite is initiated if the following criterion is satisfied:

(7) $$\frac{\varepsilon_{11}^{T}}{\varepsilon_{UTS}}\geq 1\;$$

## 4. Results and Discussion

### 4.1. Mechanical Properties of CFRP and GFRP Composites

**Figure 17:**
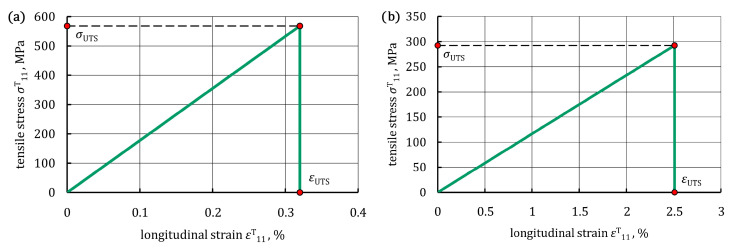
Average stress-strain curve: (**a**) single-ply CFRP composite; (**b**) single-ply GFRP composite.

Average experimental stress-strain curves for the single-ply CFRP and GFRP composites are presented in Figure 17. The stress-strain curve of the CFRP composite showed a rapid and linear stress rise and a sudden drop exceeding the ultimate strength, indicating brittleness (Figure 17a). The stress-strain curve of the single-ply GFRP composite also showed linear elastic behavior with less tensile strength and a greater strain, primarily due to the greater elasticity of glass fibers (Figure 17b). The ultimate tensile strength, *σ*_UTS_, and ultimate tensile strain, *ε*_UTS_, were easily detected since a sudden drop in the stress-strain curves was clearly seen. All the results of the tensile tests are presented in Table 9, along with the coefficients of variation.

In the current study, individual plies of the CFRP and GFRP composites were treated on the macroscopic scale as homogeneous orthotropic materials. As already mentioned, the tensile elastic modulus, $E_{11}^{T}$, was determined in the uniaxial tensile tests. The other eight material constants of the composites were predicted using the tool available in the NX Siemens software. Table 10 presents the necessary input data and nine material constants of the composites, eight of which were calculated by the software. The method of determining the input data is described in detail in Section 3.2.2. The obtained values of the material constants were used in FE analyses to define linear elastic material models of CFRP and GFRP composites.

### 4.2. Bending Behavior of 22MnB5 Grade Steel

**Figure 18:**
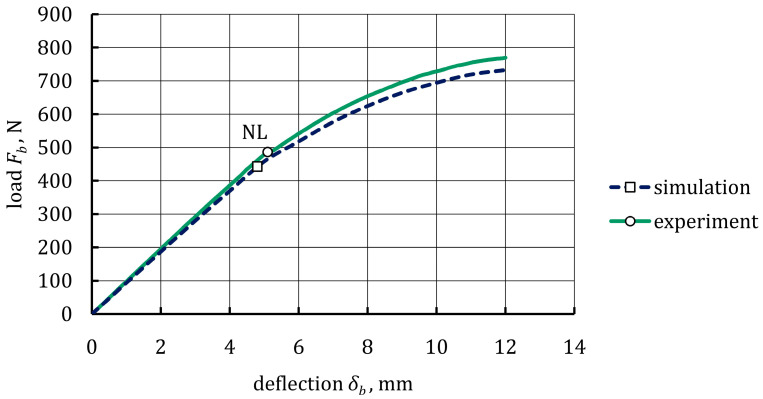
Experimental and numerical load-deflection curves for 22MnB5 grade steel specimens.

The comparison between the experimental and numerical bending response of the 22MnB5 grade steel specimens is shown in Figure 18. The average experimental load-deflection curve could be regarded as two segments. In the initial linear segment, the load (*F_b_*) increased uniformly with an increase in midspan deflection, *δ_b_*, up to point NL. It was noted that the initial linear segments were almost identical between the simulation and experimental results. Overall, the simulation and experimental curves were in good agreement (coefficient of determination, R^2^, was equal to 0.98), which confirmed the correctness of the material model of the 22MnB5 grade steel determined in the experimental tensile test.

### 4.3. Debonding Behavior between Steel and the GFRP Composite

**Figure 19:**
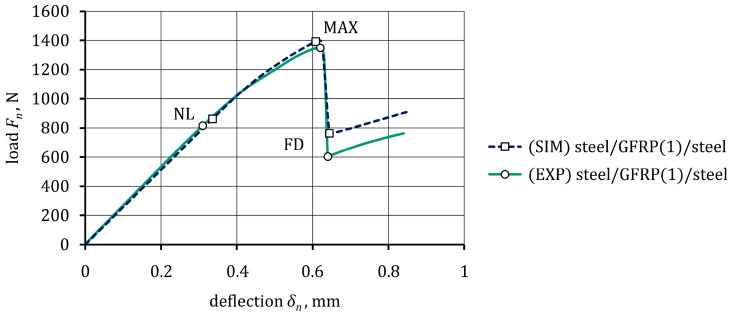
Experimental and numerical load-deflection curves from the ENF for steel/GFRP(1)/steel hybrid structure specimens.

The load-deflection curve (drawn using an average of three specimens) from the ENF test is presented in Figure 19. There were three parts to this curve, namely the linear part from zero to the NL point, the non-linear part from the NL to the MAX point, and the failure part between the MAX point and the final damage at point FD. Beyond the NL point, the curve became non-linear due to exceeding the yield strain limit of the steel. The debonding process between the steel (adherent 1) and the GFRP(1) composite began with an increasing audible crack just before reaching the MAX point. This meant that the non-linear region near the MAX point was due to the initiation and propagation of a debonding process. After the MAX point, debonding suddenly reached approximately the midspan, and a sharp drop in the load to point FD was observed.

**Figure 20:**
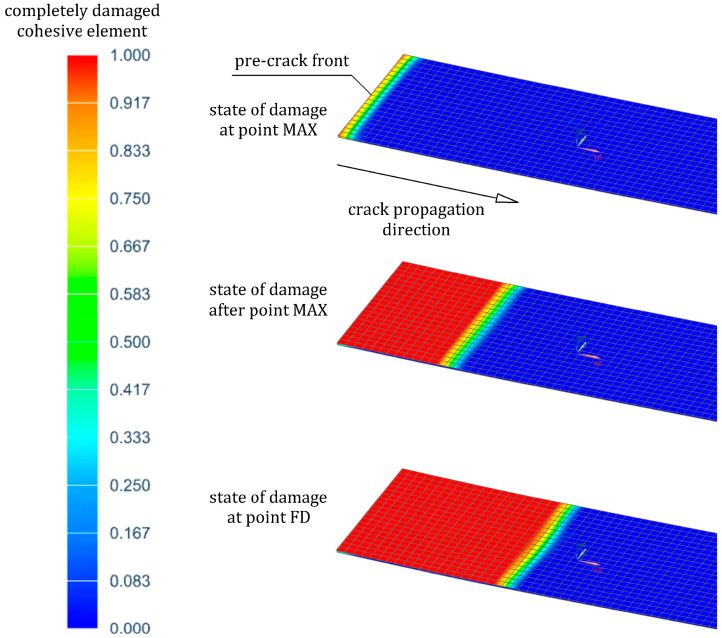
State of damage of the cohesive elements during the ENF test simulation with reference to Figure 19.

The simulation results were a valuable supplement to the experimental research. The numerical curve is compared with the experimental one in Figure 19. The former curve was also characterized by an initial elastic behavior (part zero–NL) and followed by a slight reduction in stiffness due to the plastic deformation of the steel (part NL–MAX). Simulation results of the debonding propagation in the specimen at the specific points MAX and FD are shown in Figure 20. In the cohesive zone, as expected, the crack propagated in a straight path along the cohesive elements, the plotted contour indicating the state of damage of these elements. The debonding crack initiated from the end of the pre-crack only near the MAX point. It could also be seen that debonding occurred suddenly after reaching the MAX point and propagated to the midspan, which resulted in a decreasing load (part MAX-FD). It is worth mentioning that no debonding between the GFRP(1) composite and steel (adherent 2) was observed in both the specimen and the model. The simulation values of deflection, *δ_n_*, and force, *F_n_*, at specific points NL, MAX, and FD are compared with the experimental ones in Table 11. A satisfactory agreement between the experiment and the simulation was obtained for the bilinear CZM (Figure 15c), the parameters of which are summarized in Table 12.

### 4.4. Bending Behavior of Steel/GFRP(1)/CFRP(3) Hybrid Structure

**Figure 21:**
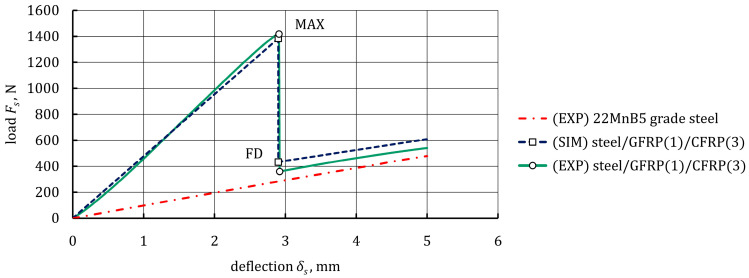
Experimental and numerical load-deflection curves from the three-point bending test for steel/GFRP(1)/CFRP(3) hybrid structure specimens.

**Figure 22:**
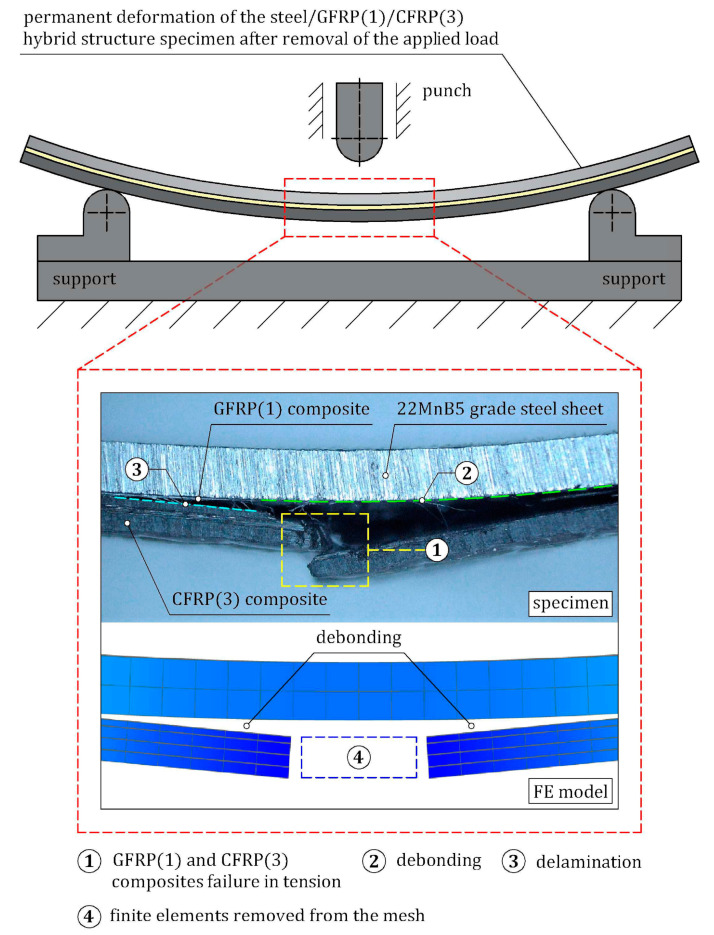
Comparison of experimental and simulated failure modes of steel/GFRP(1)/CFRP(3) hybrid structure specimens after completion of the three-point bending test.

The bending response of steel/GFRP(1)/CFRP(3) hybrid structure specimens was studied experimentally by loading, recording the force-deflection behavior, and a visual inspection of the failure modes, and also numerically using FE analysis. The average experimental and numerical load-deflection curves of hybrid structure specimens are shown in Figure 21, and a comparison of the experimental data and the numerical results for specific points is presented in Table 13. As can be seen, the numerical results were in good agreement with the experimental ones. The first part of the load-deflection curve was linear, and the hybrid structure showed a linear elastic behavior at this stage up to point MAX. After reaching point MAX, a sudden drop in load to the point FD occurred, and two main types of failure could be observed simultaneously in the specimens. The failure modes were investigated by macroscopic observation, and the damage pattern of an example specimen is shown in Figure 22. The macrograph illustrates the fracture of CFRP(3) plies after high tensile strain. Additionally, debonding is clearly shown. Based on the simulation results, it was possible to predict the sequence of occurrence of individual failure modes, which was much more difficult to identify in the experiment due to the sudden failure of the specimens. At the linear stage, all the components of the hybrid structure were in the linear-elastic range up to the MAX point, when the maximum tensile strain in the first ply of the CFRP(3) composite was reached. In the FE model, the maximum tensile strain concentration arose at the midspan, while in the area between the punch and the supports, the tensile strain concentration was much smaller. The fracture of successive layers of CFRP(3) composite occurred, the bearing capacity of the specimens then decreased, and finally, a debonded zone between the steel sheet and GFRP(1) composite was generated. It is worth mentioning that the load was still carried by the steel sheet, which exhibited strain hardening behavior.

Weight analysis of the steel/GFRP(1)/CFRP(3) hybrid structure showed that the GFRP(1)/CFRP(3) hybrid composite constituted approximately 22% of the total weight of the hybrid structure. In other words, adhesive bonding of the GFRP(1)/CFRP(3) hybrid composite to the steel sheet increased the weight of the steel sheet by approximately 28%. However, the load-bearing capacity of the hybrid structure was five times greater than that of a non-reinforced steel sheet, as shown in Figure 21. Thus, it was concluded that high weight reduction potential of steel/GFRP/CFRP hybrid structures could be obtained by an effective thickness reduction of the steel sheet with the simultaneous use of high mechanical performance GFRP/CFRP hybrid composites.

## 5. Conclusions

The FE model concept of a hybrid structure consisting of a 22MnB5 grade steel sheet, single-ply GFRP, and three-ply CFRP composites with adhesive connections between them, which enabled the examination of strength and stiffness under loading, was proposed, discussed, and experimentally validated in this paper. Carefully designed experimental procedures, including tensile tests, ENF tests, and three-point bending tests, were carried out to provide the necessary material properties of the model, which reflected the hybrid structure’s mechanical behavior under loading. The key findings of this study are listed as follows:

- The hybrid structure of a hot-formed steel sheet and GFRP and CRFP composites is a promising construction material for car body parts. Since the composite provides reinforcement in places where the part is most stressed and in the direction of the dominant stress, it will significantly increase the stiffness and strength of the part with only a slight increase in its weight. It is equally important that the hybrid steel–composite car body part designed in this way can be welded with other steel parts using well automatized assembling technology.
- The load-bearing capacity of the hybrid structure was five times greater than that of a non-reinforced steel sheet; however, its mass was only 28% greater compared to the steel sheet specimen.
- The tensile strength of the CFRP(3) composite had significant effects on the bending response of the steel/GFRP(1)/CFRP(3) hybrid structure specimens, in terms of ultimate load, debonding initiation between the steel and the GFRP(1), and stress/strain distribution in the steel.
- The experimentally verified FE model was capable of considering the effect of the mechanical properties of the individual components on the debonding behavior of the hybrid structure. This model can be used to determine the ultimate load, predict failure positions, and analyze the stress and strain distributions in steel sheets, GFRP and CFRP composites, and adhesive joints.
- To correctly determine both the composite plies and the adhesive connections between them of the hybrid structure in the proposed FE model, it was necessary to carry out a series of experimental complementary studies described in the paper.
- The galvanic corrosion-resistant steel–CFRP hybrid structure required an additional ply of GFRP composite, leading to a more complex four-phase structure. Based on the literature, the hybrid structure of a steel sheet, single-ply GFRP, and three-ply CFRP composites seems to be an original construction material. Important information about this structure can be found in this paper, but further investigations on its application as car body parts should be continued.

## Figures and Tables

**Table 1:** Chemical composition of 22MnB5 (1.5528) grade steel (wt.%) [32].

Alloying Components	C	Si	Mn	P	S	Al	N	Cr	Ti	B
min	0.21	0.15	1.10	–	–	–	–	0.10	0.015	0.0015
max	0.25	0.40	1.35	0.023	0.010	0.080	0.010	0.25	0.045	0.0040

**Table 2:** Description of the types of reinforcement used in this study.

Type of Reinforcement	Product Name	Fiber Material	Areal Weight, g/m^2^
unidirectional non-crimp carbon fiber mat	UDO^®^ UD CS 450/635	Dialead^®^ K63712	450
2 × 2 twill glass fiber fabric	Interglas^®^ 92110 FK144	warp and weft yarn: EC9-68 tex	163

**Table 3:** Selected properties of the Biresin^®^ CR122 + CH122-5 epoxy matrix system.

Modulus of Elasticity, MPa	Tensile Strength, MPa	Tensile Elongation at Break, %	Density, g/cm^3^
2800	84	5.6	1.16

**Table 4:** Chemical composition of S235JR (1.0038) grade steel (wt.%) [33].

Alloying Components	C	Si	Mn	P	S	N	Cu
content	0.17–0.20	–	1.40	0.035	0.035	0.012	0.55

**Table 5:** The nominal dimensions of the three-point bending test configuration with reference to Figure 5a (all dimensions are in mm).

Dimension	Value
total length of the specimen, *l*	100
width of the specimen, *b*	20
thickness of the specimen, *h*	1.52
span length, *s*	80
radius of the punch, *r_p_*	5
radius of the supports, *r_s_*	2

**Table 6:** The nominal dimensions of the single-ply CFRP and GFRP composite specimens with reference to Figure 6a (all dimensions are in mm).

Dimension	CFRP	GFRP
thickness, *t*	0.42 ± 0.03	0.16 ± 0.01
overall length, *l_o_*	250	
width, *w*	20	
distance between tabs, *l_s_*	150	
gauge length, *l_g_*	50	
tab thickness, *t_b_*	1	

**Table 7:** The ENF test configuration dimensions with reference to Figure 8a (all dimensions are in mm).

Dimension	Value
total length of the specimen, *l*	150
width of the specimen, *b*	20
span length, *s*	100
thickness of the adherent 1, *h*_1_	3
thickness of the adherent 2, *h*_2_	3
thickness of the GFRP(1) composite, *h_G_*	0.12
total length of the insert film, *a*	50
length of the precrack, *a*_0_	25

**Table 8:** The nominal dimensions of the three-point bending test configuration with reference to Figure 10a (all dimensions are in mm).

Dimension	Value
total length of the specimen, *l*	100
width of the specimen, *b*	20
thickness of the 22MnB5 grade steel sheet, *h_S_*	1.52
thickness of the GFRP(1) composite, *h_G_*	0.12
thickness of the CFRP(3) composite, *h_C_*	1.14
span length, *s*	80
radius of the punch, *r_p_*	5
radius of the supports, *r_s_*	2

**Table 9:** Tensile properties of the single-ply CFRP and GFRP composites.

Composite	$E_{11}^{T}$, GPa	$\sigma_{UTS}$, MPa	$\varepsilon_{UTS}$, %
CFRP	178.8 ± 6.2	568.2 ± 29.4	0.32 ± 0.03
GFRP	11.7 ± 0.4	292.5 ± 10.3	2.51 ± 0.18

**Table 10:** Input data for micromechanical analysis and material properties of CFRP and GFRP composites.

Types of Data	Constant	CFRP Composite	GFRP Composite
input data	*E_f_*, GPa	370	56
	*E_m_*, GPa	2.8	2.8
	*v_f_*, -	0.2	0.17
	*v_m_* [50], -	0.35	0.35
	*V_f_*, %	48	35
	*V_m_*, %	52	65
calculated material constants	*E*_11_, GPa	178.8	11.7
	*E*_22_, GPa	5.4	11.7
	*E*_33_, GPa	5.4	2.8
	*G*_12_, GPa	2.1	1.6
	*G*_13_, GPa	2.1	1.0
	*G*_23_, GPa	1.1	1.0
	*v*_12_, -	0.28	0.09
	*v*_13_, -	0.28	0.32
	*v*_23_, -	0.35	0.32

**Table 11:** Experimental and numerical data comparison for the ENF test of the steel/GFRP(1)/steel hybrid structure specimens.

Specific Point	*δ_n_*, mm (EXP)	*δ_n_*, mm (SIM)	Perecentage Error (*δ_n_*), %	*F_n_*, N (EXP)	*F_n_*, N (SIM)	Percentage Error (*F_n_*), %
NL	0.31	0.34	9.68	815.8	861.5	5.6
MAX	0.62	0.61	1.61	1349.5	1392.3	3.2
FD	0.64	0.65	1.56	603.1	758.4	25.8

**Table 12:** Parameters of the CZM determined in the ENF test simulation.

*K*_II(0)_, N/mm^3^	*G*_II(c)_, N/mm^3^	*σ*_II(0)_, N/mm^2^	*δ*_II(c)_, mm
4400	0.243	46	0.01

**Table 13:** An experimental and numerical data comparison for the three-point bending test of the steel/GFRP(1)/CFRP(3) hybrid structure specimens.

Specific Point	*δ_s_*, mm (EXP)	*δ_s_*, mm (SIM)	Percentage Error (*δ_s_*), %	*F_s_*, N (EXP)	*F_s_*, N (SIM)	Precentage Error (*F_s_*), %
MAX	2.91	2.90	0.34	1417.9	1384.7	2.3
FD	2.92	2.91	0.34	359.2	432.4	20.4

## Data Availability

The data presented in this study are available upon request from the corresponding authors.
